# Intrarow Uncut Weed Detection Using You-Only-Look-Once Instance Segmentation for Orchard Plantations

**DOI:** 10.3390/s24030893

**Published:** 2024-01-30

**Authors:** Rizky Mulya Sampurno, Zifu Liu, R. M. Rasika D. Abeyrathna, Tofael Ahamed

**Affiliations:** 1 Graduate School of Science and Technology, University of Tsukuba, 1-1-1 Tennodai, Tsukuba 305-8577, Japan; s2236019@u.tsukuba.ac.jp (R.M.S.); s2230267@u.tsukuba.ac.jp (Z.L.); s2136030@u.tsukuba.ac.jp (R.M.R.D.A.); 2 Department of Agricultural and Biosystem Engineering, Universitas Padjadjaran, Jatinangor, Sumedang 45363, Indonesia; 3 Department of Agricultural Engineering, University of Paradeniya, Kandy 20400, Sri Lanka; 4 Faculty of Life and Environmental Science, University of Tsukuba, 1-1-1 Tennodai, Tsukuba 305-8577, Japan

**Keywords:** uncut weeds, YOLO, instance segmentation, resource-constrained device

## Abstract

Mechanical weed management is a drudging task that requires manpower and has risks when conducted within rows of orchards. However, intrarow weeding must still be conducted by manual labor due to the restricted movements of riding mowers within the rows of orchards due to their confined structures with nets and poles. However, autonomous robotic weeders still face challenges identifying uncut weeds due to the obstruction of Global Navigation Satellite System (GNSS) signals caused by poles and tree canopies. A properly designed intelligent vision system would have the potential to achieve the desired outcome by utilizing an autonomous weeder to perform operations in uncut sections. Therefore, the objective of this study is to develop a vision module using a custom-trained dataset on YOLO instance segmentation algorithms to support autonomous robotic weeders in recognizing uncut weeds and obstacles (i.e., fruit tree trunks, fixed poles) within rows. The training dataset was acquired from a pear orchard located at the Tsukuba Plant Innovation Research Center (T-PIRC) at the University of Tsukuba, Japan. In total, 5000 images were preprocessed and labeled for training and testing using YOLO models. Four versions of edge-device-dedicated YOLO instance segmentation were utilized in this research—YOLOv5n-seg, YOLOv5s-seg, YOLOv8n-seg, and YOLOv8s-seg—for real-time application with an autonomous weeder. A comparison study was conducted to evaluate all YOLO models in terms of detection accuracy, model complexity, and inference speed. The smaller YOLOv5-based and YOLOv8-based models were found to be more efficient than the larger models, and YOLOv8n-seg was selected as the vision module for the autonomous weeder. In the evaluation process, YOLOv8n-seg had better segmentation accuracy than YOLOv5n-seg, while the latter had the fastest inference time. The performance of YOLOv8n-seg was also acceptable when it was deployed on a resource-constrained device that is appropriate for robotic weeders. The results indicated that the proposed deep learning-based detection accuracy and inference speed can be used for object recognition via edge devices for robotic operation during intrarow weeding operations in orchards.

## 1. Introduction

**Figure 1:**
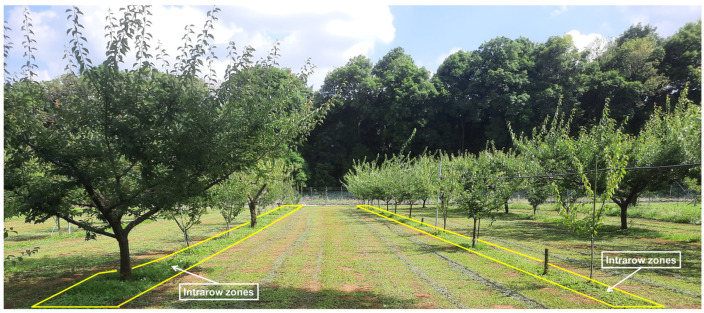
A typical orchard after practicing weeding by using a riding mower that leaves weeds in intrarow zones (T-PIRC University of Tsukuba, Ibaraki, Japan).

Interrow weeding by mechanical weeders has proven to be efficient and effective. However, mechanical weed control within tree rows (intrarows) in modern orchards presents a significant challenge. The spacing between trees within a row and the presence of obstacles (i.e., tree branches and supporting structures such as poles) often hinder the effective use of riding mowers. Since intrarow weeds cannot be reached by riding mowers, weeding in these areas must be carried out either by using hand-held and walking mowers or entirely by manual labor. Although hand-held and walking mowers are effective, these methods are time-consuming and labor-intensive and are less suitable as labor becomes more scarce due to the aging and diminishing agricultural workforce [1]. In recent practices involving weed management in orchard environments using medium-to-large machinery, however, clearing intrarow zones has been challenging (Figure 1). Weeds tend to regrow rapidly after being cut, becoming thick within 2–3 weeks. Thus, ignoring these intrarow weeds can lead to rapid growth, which competes with crops for nutrients and water and provides a conducive habitat for plant-damaging pests [2]. Therefore, there is a critical need for equipment and methods for weed control in orchards that can effectively address both interrow and intrarow areas.

In recent practices, as we mentioned above, there are some critical challenges for uncut weed at the intrarow, including the Global Navigation Satellite System (GNSS) signal interruption and precision approach to the uncut weed areas due to a lack of an accurate detection system. Even while using a mechanical mower during weeding, operators often leave weeds in the intrarow area uncut, especially around tree trunks, as they cannot differentiate between various objects. Mowing weeds manually is also difficult for the operators due to the lower tree height and the presence of support structures. To overcome these challenges, first a highly accurate detection system is required for the uncut weed and supporting poles or structures at the intrarow system in the orchards for robotic automation.

Robotics have been extensively implemented in orchard operations, including fruit harvesting, fruit tree pruning, and weed control [3]. These approaches have shown promising potential for autonomous driving of a weeder while cleaning orchard floors and could also help address the shortage of agricultural workers. However, due to the complexity of intrarow areas in orchards, the use of autonomous robotic weeders remains limited and continues to be an interesting research topic. The main challenge for robotic intrarow weeding lies in the need for a highly precise navigation system [4]. This system must allow the robot to move effectively while cutting weeds and avoiding damage to fruit trees. A navigation system is critical for an autonomous robot to determine its location and determine a path to a designated location without human intervention. Location determination relies on sensor-derived data concerning the position of the robot, while path planning involves defining a specified path for the robot to follow. This path is established using a specified control algorithm along with obstacle avoidance strategies to navigate around potential obstructions.

At present, the most common navigation system for autonomous driving is GNSS. However, when working in orchards with dense tree canopies, receivers may experience interruptions to GNSS signals, leading to a reduction in navigation accuracy and obstacle avoidance [3]. Additionally, many fruit plantations lack base stations for GNSS, making this system impractical for weeding operations [5]. Therefore, the use of other sensors, such as LiDARs, cameras, or multisensor fusion devices, is widely studied to overcome these problems. LiDAR sensors work under various light conditions, simplifying the system without requiring a powerful computer [5]. However, these methods cannot be used to differentiate many objects, which reduces their effectiveness for weeding operations in complex environments like closely spaced plants in intrarow areas. Combining these sensors with a camera can enhance the robot system’s accuracy.

**Figure 2:**
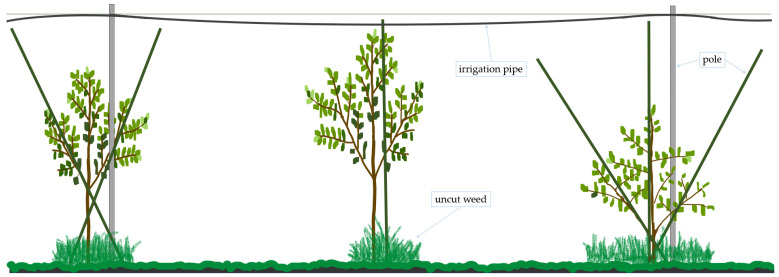
Side view of the young tree structure of young pear orchards in an intrarow area after weed control using riding mowers, where some poles were used to support tree branches and irrigation systems.

A camera can act as a sensor for detecting and distinguishing objects. This approach is necessary for intrarow robotic navigation. For example, the robot’s movement and obstacle avoidance depend on the weed locations and tree positions, respectively. Another complex challenge still exists in Japanese pear orchards. In these orchards, two or more poles are used to support young trees so they can grow upright and sturdy. Poles are used to prevent tree branches from growing downward. Poles are also commonly used to help attach ropes and irrigation pipes. However, the presence of these poles also leads to less efficient weed removal (Figure 2). Even with a hand-held weed cutter, weeds around trees are still difficult to reach. In other cases, such as modern apple orchards, a V-shaped tree architecture is deployed to produce high-quality fruit and incorporates certain poles that can serve as obstacles for autonomous navigation [6]. A camera-attached small autonomous robotic weeder can be used to easily reach weeds while avoiding the obstacles present.

Under the described conditions, object detection and localization from sensors are highly needed. For example, the location of objects can be estimated from the distance captured by a three-dimensional (3D) camera or a depth camera. A camera is also capable of distinguishing objects such as weeds and landmarks through image processing techniques [7]. The combination of deep learning algorithms with 3D cameras has made a significant contribution to object recognition, supporting robotic operations in orchards [6,8]. YOLO (You Only Look Once) is a highly efficient one-stage object detection model known for its speed, accuracy, and reliable real-time performance. YOLO has continuously improved with each release and is the preferred solution for object detection tasks, addressing both detection (object location) and recognition (object type). The bounding boxes handle object detection, while the class probability indicates the predicted object category. Therefore, the advanced capability of 3D cameras with intelligent vision systems holds significant potential for addressing the current challenges faced by conventional mechanical weeding, such as riding mowers.

Mechanical weed control using a riding mower is currently ineffective because mowers cannot reach weeds around tree trunks within a row. Therefore, an alternative mechanical method is urgently needed, such as implementing an autonomous robot capable of reaching and cleaning weeds in the area around the tree trunks. This ensures that no remaining weeds are left uncut, which could compete with fruit trees for nutrients and serve as a host for pests and plant-damaging insects. To address the weeds in these areas, an autonomous robot needs to be equipped with an intelligent vision system capable of recognizing uncut weeds and other objects, as well as weeds and object position and shape. Furthermore, vision systems can be trained under various environmental conditions, such as diverse lighting scenarios.

Therefore, the objective of this research is to develop an object recognition module using a vision system to support the robot weeder industry for intrarow weeding in orchards based on the YOLO instance segmentation algorithm. Here, we address the development of a visual detection system for uncut weed and objects to support the orchard tree in the intrarow area Furthermore, we focus on segmenting uncut weed in the intrarow area to facilitate the weeder’s easy identification of the objects and their shapes to develop datasets for deep learning models. YOLO, a single-stage deep learning model, is certainly faster in prediction than a double-stage model like Mask R-CNN, making YOLO more suitable for real-time applications on small robots in orchards [9]. Therefore, a vision system for a robotic weeder is proposed to identify three objects: uncut weeds, tree trunks, and poles. Uncut weed areas can be identified and segmented as the targeted objects for the robot’s navigation around the tree trunk. Recognized tree trunks serve as reference points for navigation, enabling precise movements such as tree-side and orbit movements around a tree trunk. The perception of tree locations also contributes to optimizing the weeding process by ensuring that the robot follows the designated intrarow paths as well as cutting weed without injuring the tree. By recognizing weeds, tree trunks, and fixed poles, robot weeders can be designed to have the capability to remove weed in the intrarow areas of orchards.

## 2. Related Works

Recent advancements in sensors, instrumentation, and computing technologies have offered significant opportunities to improve in-row weeding operations by providing reliable mechanical actuators for removing weeds without damaging the main crop [10]. These technologies are increasingly employed to address two primary challenges in autonomous orchard weeding systems: first, achieving effective and efficient weed removal across the entire orchard floor, including areas around tree trunks; and second, enabling autonomous movement throughout the orchard [4,11]. Moreover, conventional control methods, such as herbicide application in vine rows and the areas between them (middles) or a combination of herbicide strip application in vine rows and cutting weeds in the middle tree rows [12], should be minimized due to environmental and health concerns.

The use of robotic technology for intrarow weeding operations in orchards has advanced significantly. An automatic GNSS-based intrarow weeding machine with a cycloid hoe was developed [13], utilizing real-time kinematic (RTK)-GNSS for navigation and control. Additionally, an autonomous electric robot equipped with a 2D laser scanner and sonar sensor for intrarow weed management in vineyards has been introduced [14]. This robot can autonomously navigate between tree rows, cutting intrarow weeds with the adjustable side-shift movement of the rotary weeder without injuring the trees. However, the GNSS signal may be obstructed when applied to small robots obscured under tree canopies. Another method uses a robotic lawn mower (RLM) with the capability to navigate in any direction. Hossain and Komatsuzaki (2021) reported that the RLM outperforms conventional mowers in small orchards, although addressing intrarow weeds around trees remains a challenge [15]. Therefore, another sensor, such as a vision sensor, should be considered to overcome these potential problems.

Astrand and Baerveldt (2002) investigated the use of a vision sensor for weeding operations by employing two vision systems to guide a robot along the crop row and distinguish between crops and weeds [16]. The vision system was only tested in indoor operations, such as in greenhouses that have constant illumination. Subsequently, intrarow weeding operations in orchards based on vision using a stereo camera system have also been designed, primarily focused on trunk detection [17]. However, weed location, which is less efficient for intrarow weeding, has not been considered in these systems. The use of recent camera technology and image processing techniques shows promise in addressing intrarow weed control challenges [18]. Ground-based robots equipped with RGB and depth cameras have been employed for automated weed removal through targeted herbicide spraying or mechanical in-row removal [19,20]. However, most of the vision systems utilized were not trained, which may lead to failure when illumination is insufficient. Therefore, intelligent vision systems such as deep learning [21], which are pretrained even in the presence of limited illumination, demonstrate satisfactory accuracy.

Sapkota et al. (2022) employed Mask R-CNN, a powerful convolutional neural network deep learning algorithm, to detect and segment weeds in cotton plants [22]. However, the YOLOv8 has better accuracy and faster inference time in instance segmentation compared to Mask R-CNN [23]. Several deep learning algorithms, such as YOLOv5, YOLOv7, and YOLACT, are effective at detecting and masking objects [24] and have the potential to delineate remaining weed areas around trees that cannot be reached by weeding mowers. However, research on weed recognition in orchards via segmentation has been limited in the literature, especially for intrarow uncut weeds after weeding operations via conventional mechanical weeders or robotic operations. Therefore, deep learning segmentation techniques [25] can be effective and powerful for detecting and estimating the area of uncut weeds in orchards and implementing new vision systems for weeding operations on robotic platforms.

## 3. Materials and Methods

This study focused on addressing the intrarow weeding challenge in orchards by developing an image processing module that leverages YOLO instance segmentation algorithms. The algorithm can be utilized to recognize weeds and obstacles, providing support for an autonomous weeder robot during intrarow weeding operations. To achieve this goal, several steps were taken. In the following section, the original data extracted from the video acquired from the orchard were manually labeled, and polygon-shaped bounding boxes were used to annotate object classes such as uncut weeds, tree trunks, and poles for subsequent training and testing. YOLOv5 and YOLOv8 for instance segmentation were subsequently trained on the training dataset and applied to detect the aforementioned multiclass objects. Finally, an assessment was conducted to evaluate the performance of the trained model.

### 3.1. Image Acquisition

**Figure 3:**
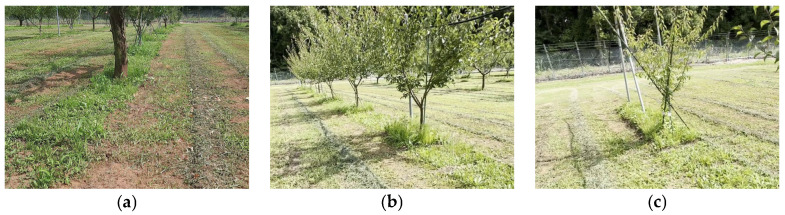
A Japanese pear fruit orchard after weeding by riding mower. (**a**) Uncut weeds around a mature tree; (**b**,**c**) indicate the remaining weeds (which a riding mower cannot reach).

Japanese pear orchard images were taken at Tsukuba-Plant Innovation Research Center (T-PIRC), University of Tsukuba, Ibaraki, Japan, on 20 June 2023, after a weeding operation was conducted using a weeding mower. Videos were recorded with two smartphone models (Samsung Galaxy A51; Samsung Electronics Co., Ltd., Suwon, Korea; and iPhone 14; Apple, Inc., Cupertino, CA, USA) at a resolution of 1920 × 1080 pixels, positioned approximately 50 cm above the ground while walking between tree rows. The data collection simulated the perspective of a camera attached to the robotic weeder moving among the orchard’s interrow trees. Approximately 975 images were extracted from the recorded videos and saved in portable network graphics (PNG) format, capturing orchard floor conditions after the weeding operation (Figure 3). The images depict uncut weeds, orchard trees, and fixed poles for young trees and irrigation pipes.

### 3.2. Image Dataset

**Figure 4:**
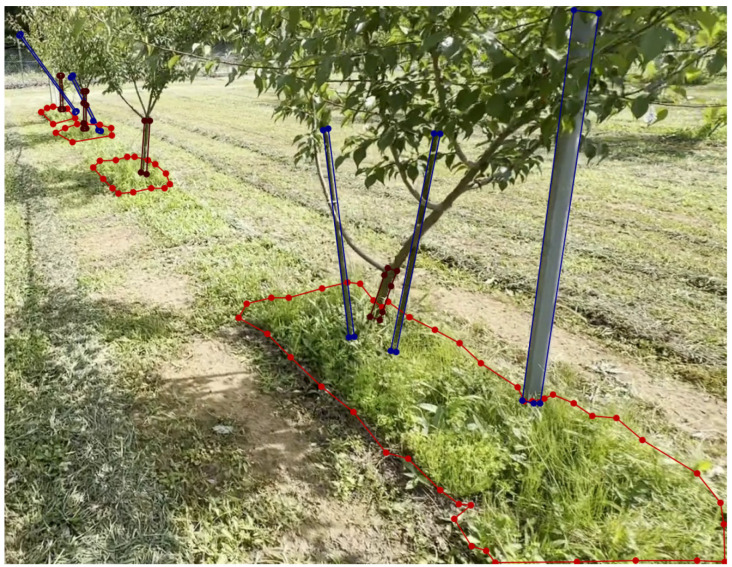
Polygon labeling for YOLO instance segmentation of young pear trees: uncut weeds (red), tree trunks (brown), and poles (blue).

The obtained images were augmented through flipping and cropping to various sizes, with a maximum of 640 × 480 pixels, to optimize the training and testing process and ensure compatibility with the edge-device camera resolution. Data augmentation, which is crucial for state-of-the-art deep learning systems, helps to increase accuracy [26]. The final image set (5000 images) after augmentation was manually annotated using Labelme (https://github.com/wkentaro/labelme, accessed on 10 July 2023). After that, polygonal bounding boxes were drawn, marking objects such as uncut weeds (red), tree trunks (brown), and fixed poles (blue). Red indicates uncut weed areas, brown represents the main tree trunks, and blue represents both plastic and iron poles. An example of an annotated image for YOLO-based instance segmentation is shown in Figure 4. The annotated images were stored in JSON format and later converted to TXT format because YOLO does not support the JSON format.

**Figure 5:**
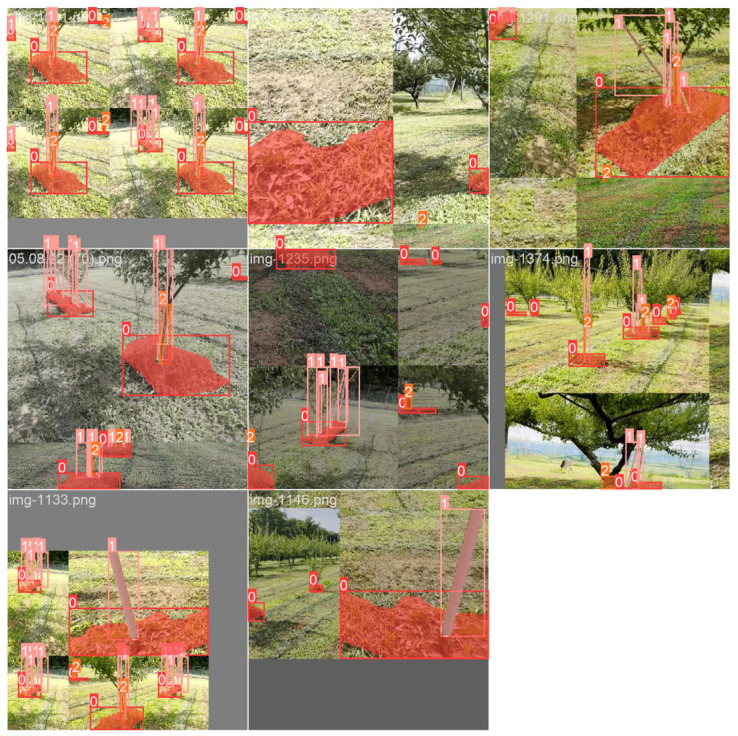
Example of online augmentation (HSV adjustment, scaling, translation, and mosaicking) and its associated annotation for an eight-batch training dataset with a 640 × 640 image size. The numbers 0, 1, and 2 represent weed, pole, and tree trunk classes, respectively.

After labeling, the image dataset (5000 images) was randomly divided into a training set (4000 images) and a testing set (1000 images). Subsequently, the training set was further augmented via hue, saturation, value (HSV) adjustment, scaling, translation, and mosaicking to enhance the generalizability of the trained models and minimize the risk of overfitting. The augmentation could fulfill data requirements not only obtained during daylight but also under various light conditions, angles, and distances. This augmentation, also referred to as online augmentation (Figure 5), was performed with the Ultralytics library.

### 3.3. Network Architecture

We utilized recent versions of the YOLO model, YOLOv5, and YOLOv8 [27,28]. These models have an instance segmentation feature, extending the object detection task to predict both the object and its shape by segmenting objects from the detected background. Before deciding to use YOLOv5 and YOLOv8, we considered other models that have capabilities in instance segmentation, such as Mask R-CNN [29]. However, we selected YOLOv5 and YOLOv8 for several reasons. First, the YOLO family has strong performance in object detection tasks, making them competitive and suitable for our instance segmentation tasks. Second, both model algorithms were developed using PyTorch, a widely used open-source framework in the extensive deep learning community. This framework has simplified the process of training and testing with a customized dataset. Third, YOLOv8 outperformed Mask R-CNN in the benchmark paper by detecting, segmenting, and classifying specific objects. We also compared the performance of the trained models to determine the most efficient and low-computational-cost model for use in robotics in real-time orchard operations.

#### 3.3.1. YOLOv5 Instance Segmentation

**Figure 6:**
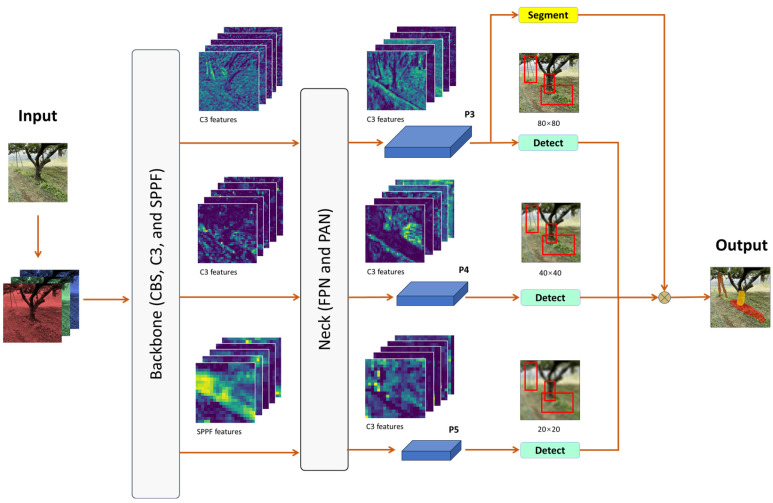
Network structure of the YOLOv5 instance segmentation for weed, tree trunk, and pole recognition in orchards.

YOLOv5 instance segmentation (YOLOv5-seg) is an extension of YOLOv5 version 7.0 designed for object detection with an additional segmentation feature in the head. The networks consist of a backbone, neck, and head [30], as illustrated in Figure 6. The backbone is responsible for extracting features from the input image and comprises multiple CBS (convolution, BN layer, and SiLU activation function), C3 (concentrated comprehensive convolution), and SPPF (spatial pyramid pooling-fast) layers to extract essential features from input images. The neck combines and integrates the feature pyramid network (FPN) and the path aggregation network (PAN) to achieve multiscale feature fusion of the features extracted through the backbone to obtain rich feature information.

The head of YOLOv5-seg consists of two branches: the object detection head and the instance segmentation head. The object detection head inherits the multiscale feature fusion mechanism of the regular YOLOv5 object detection model and detects objects of different sizes at three feature scales of 20 × 20, 40 × 40, and 80 × 80. On the other hand, the instance segmentation head achieves pixel-by-pixel classification prediction and generates binary masks for the object through a small, fully convolutional neural network (FCN). Finally, the outputs from both heads are combined to produce instance segmentation outputs (Figure 6).

YOLOv5-seg utilizes the Ultralytics algorithm, namely, AutoAnchor, which assesses and adjusts anchor boxes if they are inappropriately sized for the dataset and training [31]. The YOLOv5-seg model is classified into five sizes based on complexity: YOLOv5n-seg (nano), YOLOv5s-seg (small), YOLOv5m-seg (medium), YOLOv5l-seg (large), and YOLOv5x-seg (extralarge). As the model complexity increases, the accuracy improves, while the inference speed and model size decrease.

#### 3.3.2. YOLOv8 Instance Segmentation

**Figure 7:**
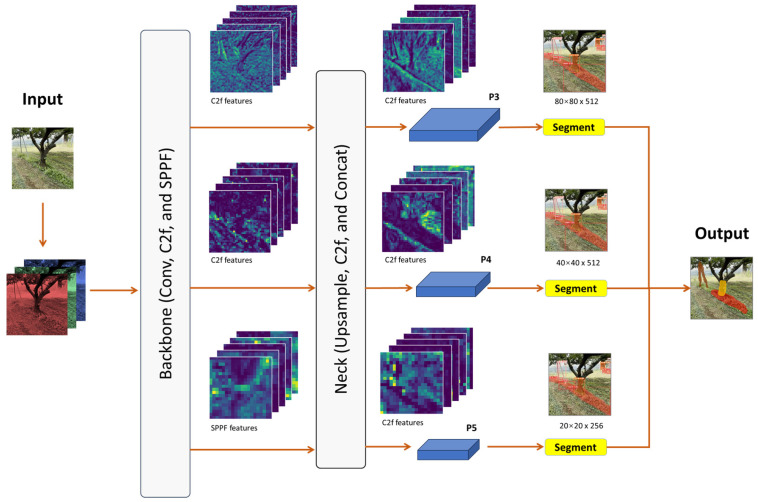
Network structure of YOLOv8 instance segmentation for weed, tree trunk, and pole recognition in orchards.

YOLOv8 features an instance segmentation model named YOLOv8-seg, which applies principles inspired by YOLACT [32] for instance segmentation. The structure of YOLOv8-seg consists of three main parts: the backbone, neck, and segmentation head [33] (Figure 7). The backbone of YOLOv8-seg initiates feature extraction from an image using a modified CSP layer known as the C2f module. This module acts as a cross-stage bottleneck that incorporates a convolutional layer to enhance detection accuracy by integrating high-level features. Then, the SPPF layer extracts information from images at varying scales, which significantly enhances the model’s generalization capabilities. The neck is similar to that of YOLOv8, a feature pyramid network (FPN), which integrates diversely sized features. The head consists of detection and segmentation branches. The detection branch outputs the category and bounding box, while the segmentation branch outputs the *k* prototypes along with *k* mask coefficients. Both detection and segmentation are computed in parallel in the segmentation head, which produces segmentation outputs at various scales inherited from the previous part (neck). Finally, the segmentation outputs are combined to produce a single output, for instance, segmentation, as shown in Figure 7.

In contrast to YOLOv5-seg, YOLOv8-seg employs an anchor-free model with a separate head to independently process object, classification, and regression tasks. This design enables each branch to focus on its specific task and enhances the model’s overall accuracy. There are five types of YOLOv8-seg based on model complexity. In this study, we utilized YOLOv5-seg and YOLOv8-seg models of nanoscale and small types due to their compact and lightweight design. These characteristics make them suitable for real-time application in orchards, particularly on resource-constrained devices. All the models were then trained using our custom dataset and tested for their ability to recognize uncut weeds, tree trunks, and fixed poles in the intrarow area of pear orchards.

### 3.4. Systems for Network Training and Testing

Network training was conducted based on the deep learning framework PyTorch version 2.1 on a computer equipped with an Intel Core i3 9th Gen CPU, 16 GB of RAM, and an NVIDIA GeForce GTX 1050 Ti 4GB GPU (768 CUDA cores) running on a Windows 11 64-bit operating system. Software tools, including CUDA 11.8, OpenCV 4.8.0.74, YOLOv5 version 7.0, and YOLOv8 from Ultralytics 8.0.200, were used for multiclass object segmentation training based on the PyTorch framework. In general, the training configurations of both YOLOv5-based and YOLOv8-based instance segmentations, such as the input size, batch size, learning rate, momentum, decay, epoch, and class number, can be found in Table 1. We opted for smaller versions of the n and s variants to conduct our studies, considering their efficiency and cost-effectiveness for resource-constrained devices in real-world applications. Following training, all the models were also tested on a single-board computer, the NVIDIA Jetson Xavier NX 8GB, which features a 6-core arm64 CPU (384 CUDA cores) and an Ubuntu 20.04 operating system that has the capability to be attached to robots for outdoor applications.

### 3.5. Evaluation Metrics

The performance of YOLO instance segmentation for weeds and landmarks in orchard environments was evaluated in terms of detection accuracy, model complexity, and inference time [34], which are detailed below:

#### 3.5.1. Detection Accuracy

Several common metrics for object detection and image segmentation, including precision (P), recall (R), and mean average precision (mAP), were used to assess trained models on test data [24,35]. The mean average precision (mAP) was used as the main evaluation metric, especially the mAP50. The mAP was derived from the intersection-over-union (IoU) or Jackard index, which represents the average of the precision values at various recall levels for a specific IoU threshold. The IoU is used to measure the similarity between two regions and is defined as the ratio of intersection to union of both. The metric equations used are indicated in Equations (1)–(3) as follows:

(1) $$IoU(A,B)=\frac{\left|A\cap B\right|}{\left|A\cup B\right|}$$

(2) $$Precision=\frac{True\;Positives}{True\;Positives\;+\;False\;Positives}$$

(3) $$Recall=\frac{True\;Positives}{True\;Positives\;+\;False\;Negatives}$$

In general, the average precision (AP) for a single class is determined by ranking the model’s predictions by their confidence scores and then calculating the area under the precision-recall curve, as indicated in Equation (4).

(4) $$AP=\sum_{n}\left({Recall}_{n}-{Recall}_{n-1}\right).{Precission}_{n}$$

Since our research was intended to detect three classes, the mAP was equal to the average AP of each class. The mAP50 refers to the AP at an IoU threshold of 0.5. The mAP50:95 refers to the AP for each IoU threshold from 0.5 to 0.95 in increments of 0.05; those values are then averaged as indicated in Equation (5):

(5) $$mAP=\frac{{AP}_{IoU=0.5}+{AP}_{IoU=0.55}+\cdots +{AP}_{IoU=0.95}}{k}$$

where k is the number of IoU thresholds considered.

The mAP50 and mAP50:95 were used to evaluate the ability of the trained model to accurately segment objects at varying IoU thresholds. Among the metrics, the mAP was the primary metric in multiclass object detection for evaluating our trained models. Furthermore, mAP, precision, and recall were used to evaluate both the bounding boxes and segmentation masks of the instance segmentation models.

#### 3.5.2. Number of Model Parameters

The number of model parameters was used to measure the complexity of the model. Models with a large number of parameters usually require more memory for deployment, and vice versa. In addition, the number of model parameters affects the computational cost and inference time.

#### 3.5.3. Computational Cost and Inference Time

The computational cost refers to the number of computations expressed in floating-point operations (FLOPs) required by a model. The FLOPs serve as a metric for measuring the computational load of a model and indicate the number of operations required for a single instance. In real-time applications, this factor is crucial. On the other hand, inference time is the duration that a trained model uses to make predictions from the given input image. The inference time varies depending on the computing hardware used. The inference time was calculated as the average time required to predict all the test set images for each YOLO model. In contrast to FLOPs, the inference time was hardware-dependent. To comprehensively assess the model’s efficiency in a real orchard environment, which enables robots to recognize weeds and obstacles, we considered both training and inference times. Additionally, videos were prepared to complete the assessment of the trained models, providing a holistic evaluation of their performance in practical scenarios.

## 4. Results

### 4.1. Training Details

**Figure 8:**
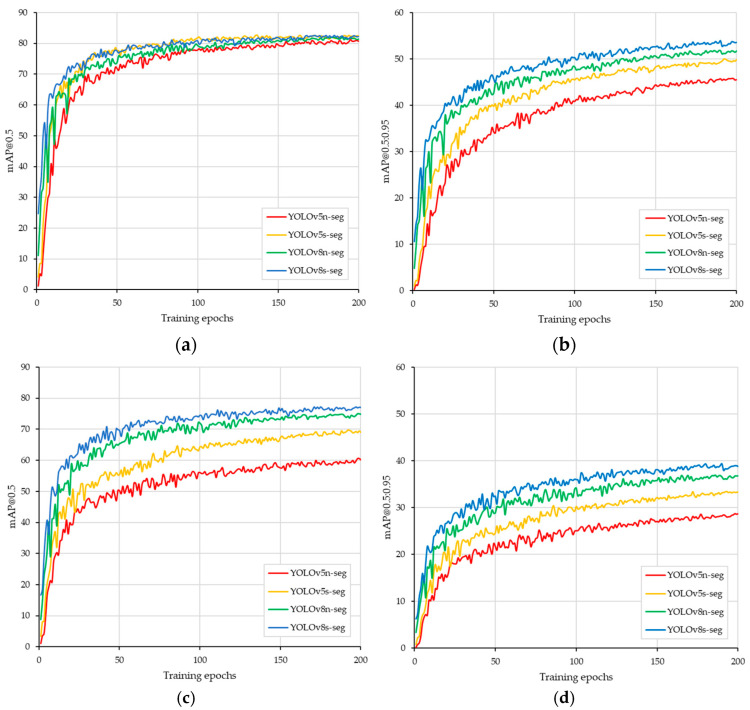
Training curve of four YOLO instance segmentations. (**a**) mAP@0.5 of the box, (**b**) mAP@0.5:0.95 of the box, (**c**) mAP@0.5 of the mask, and (**d**) mAP@0.5:0.95 of the mask.

Figure 8 shows the training curves for mAP@0.5 and mAP@0.5:0.95 for the four instance segmentation models in terms of object detection and segmentation accuracy. In general, these models demonstrated potential in terms of high convergence and detection accuracy, although their segmentation accuracy may not surpass their detection accuracy. An mAP@0.5 was detected within approximately 80% of the epochs (Figure 8a), while YOLOv8n-seg and YOLOv8s-seg had detection accuracies greater than 50% (mAP@0.5:0.95) at the same epochs (Figure 8b). According to the segmentation training curve, there were differences between YOLOv5 and YOLOv8. After 50 epochs, the mAP@0.5 of all the models increased gradually until the end of the epochs. The accuracies of YOLOv5n-seg and YOLOv5s-seg were less than 70%, while those of YOLOv8n-seg and YOLOv8s-seg reached more than 70% (Figure 8c). The same pattern also occurred in the mAP@0.5:0.95 segmentation, where the YOLOv8 models always had a greater mAP@0.5:0.95 than the YOLOv5 models (Figure 8d). The training curve also confirmed that 200 epochs of training were sufficient for real-time application in orchard intrarow weed detection.

### 4.2. Testing Results

Table 2 summarizes the performances of the four YOLO instance segmentation models. Overall, all the models demonstrated good accuracy in detecting and segmenting three types of objects: uncut weeds, tree trunks, and fixed poles. For detection, the mAP@0.5 values ranged from 80.90%, achieved by YOLOv5n-seg, to 82.40%, achieved by YOLOv8s-seg. Moreover, the mAP@0.5:0.95 values ranged from 45.80% for YOLOv5n-seg to 53.90% for YOLOv8s-seg. In terms of segmentation masks, the mAP@0.5 and mAP@0.5:0.95 of the YOLOv8s-seg model were the most accurate compared to those of YOLOv5n-seg, YOLOv5s-seg, and YOLOv8n-seg. The lower value of mAP@0.5:0.95 compared to mAP@0.5 was attributed to the former using higher IoU thresholds for mAP calculations, which apply more stringent criteria.

**Figure 9:**
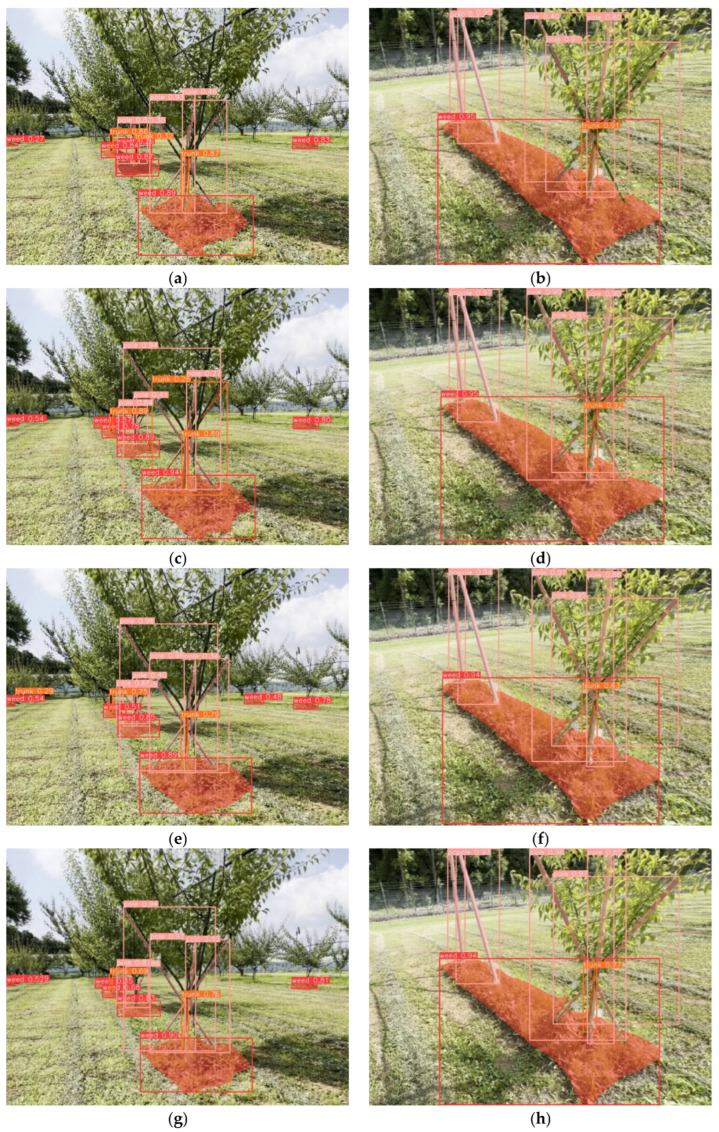
Detection of weed removal areas using custom-trained models: (**a**,**b**) YOLOv5n-seg, (**c**,**d**) YOLOv5n-seg, (**e**,**f**) YOLOv8n-seg, and (**g**,**h**) YOLOv8s-seg. The bounding boxes and masks with the same color represent the same class. The red, orange, and pink colors represent the detection and segmentation of uncut weeds, tree trunks, and poles, respectively.

Figure 9 shows examples of images predicted by the four trained YOLO instance segmentation models. The models produced visually good predictions for images with diverse backgrounds in a real orchard, including the middle and edge of tree rows. Furthermore, the predictions were also tested on videos with a resolution of 1920 × 1080 pixels. Overall, these results illustrated the efficacy of YOLO instance segmentation models for recognizing uncut weeds, tree trunks, and poles.

To examine the differences in performance among the four YOLO instance segmentation models, an average accuracy was calculated by averaging the results obtained across different model variants within each instance segmentation type (Table 2). Overall, in terms of segmentation, YOLOv8s-seg achieved the best mAP@0.5 and mAP@0.5:0.95 at 76.70% and 39.30%, respectively. In terms of detection, YOLOv8s-seg and YOLOv5s-seg exhibited high accuracy, having similar mAP@0.5, while the mAP@0.5:0.95 of YOLOv8-seg surpassed that of YOLOv5-s. Therefore, in this case, YOLOv8s-seg is considered the most accurate instance segmentation model among the four instance segmentation models used in this study. Although average accuracy may not be a rigorous metric for comparing the different types of YOLO instance segmentation, their differences suggested that YOLOv8-based instance segmentation performed better than YOLOv5-based segmentation.

Model complexity and inference time are important for practical deployments in resource-constrained settings. Table 3 shows the number of model parameters, GFLOPs, inference times, FPS, and mean averages of the four YOLO instance segmentation models. The GFLOPs and inference times tended to increase linearly with the number of model parameters. YOLOv8s-seg seemed to have the highest GFLOPs, with the longest inference time of 43.88 milliseconds (ms). In contrast, YOLOv5n-seg was the most computationally efficient and the fastest in inference. However, there were likely trade-offs in model selection between accuracy and inference time. As shown in Table 3, the average precision of segmentation tended to be inversely proportional to the inference time. The faster the inference time, the lower the precision obtained.

Table 4 shows the results of the model inference for a 15 s video file in mp4 format. All the models could process videos effectively on a PC with a GPU, with varying FPS depending on the type of YOLO instance segmentation model. However, generally, the YOLOv5-based model had a greater FPS than the YOLOv8-based model, and the FPS decreased linearly with the number of parameters and number of GFLOPs. The same test configuration was used on the Jetson platform, although the FPS on the Jetson platform was approximately half that on a PC. Overall, all the YOLO instance segmentations performed in this study have the potential to be deployed as edge devices for real-time weed identification, tree trunk identification, and fixed pole recognition based on segmentation at approximately 11 to 23 FPS.

The results clearly showed that all YOLO instance segmentations were used for detection, and an mAP@50 of approximately 80% was achieved. The YOLOv8-based models exhibited better segmentation accuracy than the YOLOv5-based models. The number of parameters and GFLOPs were inversely proportional to the inference speed, with nano-sized models surpassing their small-sized models. From these results, we indicate YOLOv8n-seg as the most efficient model suitable for object detection because of the segmentation needs of robotic weeders.

## 5. Discussion

Research on weed segmentation for distinguishing weeds and main crops in agronomy and horticulture is common. The weeds are typically identified as individual objects rather than areas [36,37]. However, there is a gap in the literature regarding weed recognition in orchard soils, particularly regarding differences between cut and uncut weeds, including their respective areas during intrarow weeding operations. Additionally, manual or hand-held weed cutters and chemical spraying still cause ergonomic and environmental concerns. This study makes a unique contribution to the research community by addressing the ability of deep learning to perform weeding operations in uncut areas in orchards for real-time application. Moreover, this research introduced a novel approach for recognizing uncut weeds, tree trunks, and poles based on YOLO instance segmentation and achieved excellent performance characterized by high segmentation accuracy and fast inference times.

**Figure 10:**
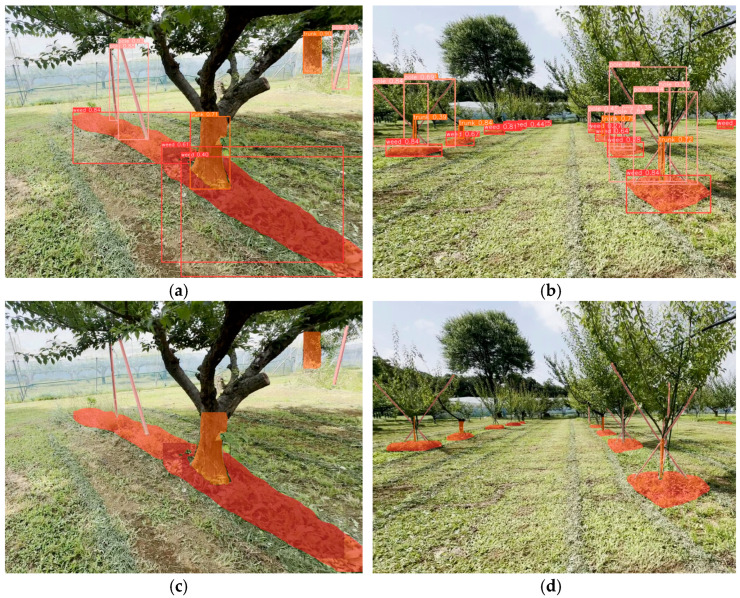
YOLOv5n-seg detection results: (**a**,**b**) with bounding boxes and confidence information and (**c**,**d**) without bounding boxes and confidence information.

Our trained YOLO instance segmentation model could detect both the location and shape/area of an object. Information about weed areas, the form of fruit tree trunks, and poles can be used to support the operation of an autonomous robotic weeder system. The robotic weeder can recognize the remaining weeds in an orchard and can be designed to move toward the location of the weeds. Even for mature trees without structural support, such as poles, diameter information is used to guide the tree-oriented circular movement of robot weeders in interrow areas [38]. Figure 10 shows examples of segmentation objects obtained using custom-trained YOLOv8n-seg, with the confidence set at 0.25. Uncut weeds, tree trunks, and poles were clearly detected and segmented well in close proximity to the camera sensor. However, objects at considerable distances were not all detected accurately. However, for overlapping objects, such as intersecting poles and poles behind the trees, YOLOv8n-seg demonstrated effective segmentation. The models were tested on limited computing devices, such as the Jetson Xavier NX. The custom-trained YOLOv8n-seg was deployed and was capable of performing instance segmentation at 16 FPS on a 640 × 640 image size. That model also performed faster when the input image was changed to 320 × 320. This means that the model can be applied to robotic platforms in real time.

This study has several limitations that require further improvement. Although our study did not focus on detection accuracy surpassing YOLOv5-based compared to YOLOv8-based methods, further research can be carried out for modifications to the YOLOv8n-seg model architecture, such as in the head, to achieve greater detection and segmentation. Objects located far from the camera, such as the tree trunk and poles in Figure 10d, were not well segmented. Therefore, the annotation process for small objects should be intensified and clear. The trained models have been tested for predicting objects in short videos, but their performance in handling long videos remains unexplored. The current research demonstrates the efficacy of a single-stage transformer-based video instance segmentation framework for efficiently segmenting instances in complex real-time computations such as long video [39]. That model utilized parameters three times bigger than YOLO’s small variants. Therefore, the next study can focus on comparing and modifying the model employed in this research. The accuracy achieved in this study is localized to the orchard area for which the data were utilized in training. To enhance accuracy and practical applicability, the model can be further trained with more diverse data obtained from other orchard environments. Another deep learning method, such as learning discriminative features, also has the potential to be employed; however, this method requires a larger dataset [40]. Colors, areas, distances, diameters, and lengths of objects in an orchard are potential features to improve the accuracy of detection. Additionally, to maintain inference speed on the single-board computer, the trained model currently uses the PyTorch framework, which can be converted to other formats, such as ONNX or TensorRT, which is a common and lightweight framework for edge devices capable of improving inference speed three to five times faster than PyTorch [41]. After accelerating inference, the input resolution can be increased to enhance the quality of object recognition by robotic weeders during operations in orchards.

**Figure 11:**
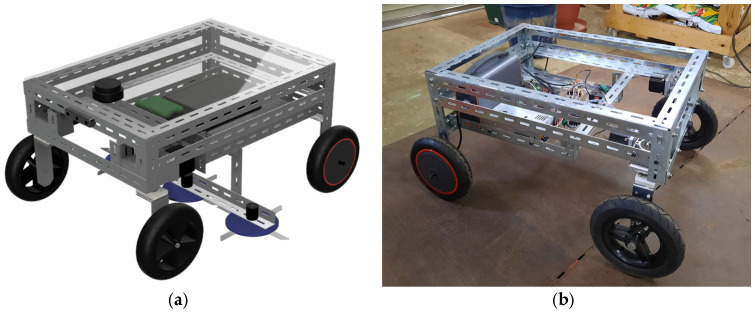
The autonomous robotic weeder preparation for the orchard, especially at the intrarow area: (**a**) 3D model of the final product and (**b**) robotic weeder in progress during reconstruction.

In the next step, the trained model on an edge device will be attached to an autonomous robotic weeder (Figure 11), enhancing its object detection and navigation system for weeding operations in an orchard. During real-time operations, a depth camera provides input data to the detection module. The detected objects (weeds, tree trunks, and obstacles) will be processed to extract required features such as area, distance, and diameter. This extracted information is then utilized for the steering and driving system of the robot, as well as controlling the weed cutter. The robotic flatform has dimensions of 70 × 50 × 43 cm (length × width × high), while the cutter is set wider than the robot to effectively clean the weed during turning in intrarow areas. A 360-degree LiDAR is also equipped to avoid obstacles.

## 6. Conclusions

Object recognition by robotic weeders is important for weeding operations in orchards. In this study, we introduced a novel training dataset for object recognition based on YOLO instance segmentation. We used four YOLO algorithm models (YOLOv5n-seg, YOLOv5s-seg, YOLOv8n-seg, and YOLOv8s-seg) suitable for resource-constrained devices and then selected the most effective model for testing on the edge device in the autonomous weeder system. The training was conducted using a similar configuration. The custom-trained models were tested using images and videos from advanced computers and edge devices, such as the Jetson Xavier NX. Therefore, the following points are outlined to conclude the research results:

1. The vision system could recognize uncut weeds within rows for robotic platforms. For recognition, the YOLO-based model was used, and several deep learning networks were evaluated for satisfactory recognition using instance segmentation.
2. According to our findings for orchard object recognition, using instance segmentation, YOLOv5-based models were faster at prediction but exhibited less accuracy. In contrast, the YOLOv8-based models demonstrated higher accuracy, with speeds not significantly different than those of the most accurate YOLOv5 model.
3. YOLOv8n-seg was the most efficient and accurate method for recognizing weeds remaining between rows and obstacles; this approach is fast and highly accurate compared with the YOLOv8 family because of its lower computational cost. Therefore, this model can be used for weeding operations in intrarow areas as a second operation after riding mower weeding that cannot reach weeds around tree trunks.
4. This approach can be adopted in edge devices for in-field operations for autonomous robotic weeders.

Therefore, further experiments will be conducted using edge devices installed on a robotic platform for intrarow weeding operations.

## Figures and Tables

**Table 1:** Network parameters of YOLOv8 instance segmentation.

No.	Model	Image Size	Batch	Learning	Momentum	Decay	Epoch	Class
1	YOLOv5n-seg	640 × 640	16	0.01	0.937	0.0005	200	3
2	YOLOv5s-seg	640 × 640	8	0.01	0.937	0.0005	200	3
3	YOLOv8n-seg	640 × 640	16	0.01	0.937	0.0005	200	3
4	YOLOv8s-seg	640 × 640	8	0.01	0.937	0.0005	200	3

**Table 2:** Object instance segmentation performance of four YOLO instance segmentation models on the testing dataset (precision, recall, mAP@0.5, and mAP@0.5:0.95) in percentages.

No.	Model	Box				Mask			
		Precision	Recall	mAP@0.5	mAP@0.5:0.95	Precision	Recall	mAP@0.5	mAP@0.5:0.95
1	YOLOv5n-seg	80.70	75.90	80.90	45.80	69.90	60.60	60.60	28.70
2	YOLOv5s-seg	**83.50**	**78.20**	82.10	50.00	76.40	67.40	69.70	33.40
3	YOLOv8n-seg	82.50	75.50	81.17	51.70	77.10	70.50	74.60	36.90
4	YOLOv8s-seg	81.20	78.10	**82.40**	**53.90**	**79.50**	**71.50**	**76.70**	**39.30**

Note: The best results are marked in bold.

**Table 3:** Number of parameters, GFLOPs, inference times, and segmentation mAPs of the trained models (GFLOPs stands for giga floating-point operations (FLOPs), with 1 GFLOP being equal to 109 FLOPs; and mAP denotes the mean average precision of segmentation).

No.	Model	Parameters (Millions)	GFLOPs	Inference Times (ms)	mAP@0.5	mAP@0.5:0.95
1	YOLOv5n-seg	1.88	6.70	25.90	60.60	28.70
2	YOLOv5s-seg	7.40	25.70	29.90	69.70	33.40
3	YOLOv8n-seg	3.26	12.10	36.92	74.60	36.90
4	YOLOv8s-seg	11.79	42.70	43.88	76.70	39.30

**Table 4:** Inference results of YOLO instance segmentation on advanced and resource-constrained devices for deep learning.

No.	Model	GeForce GTX 1050Ti (FPS)	Jetson Xavier NX (FPS)
1	YOLOv5n-seg	48.30	23.75
2	YOLOv5s-seg	42.37	17.01
3	YOLOv8n-seg	40.48	16.67
4	YOLOv8s-seg	24.14	11.10

## Data Availability

The dataset that was generated and analyzed during this study is available from the corresponding author upon reasonable request, but restrictions apply to data reproducibility and commercially confident details.
